# Dynamics of *Cardicola* spp. Infection in Ranched Southern Bluefin Tuna: First Observation of *C. orientalis* at Transfer

**DOI:** 10.3390/pathogens12121443

**Published:** 2023-12-13

**Authors:** Melissa J. Carabott, Cecilia Power, Maree Widdicombe, Kirsten Rough, Barbara F. Nowak, Nathan J. Bott

**Affiliations:** 1 School of Science, RMIT University, Melbourne, VIC 3083, Australia; s3722648@student.rmit.edu.au (M.J.C.); cecilia.power@rmit.edu.au (C.P.); s3599119@student.rmit.edu.au (M.W.); b.nowak@utas.edu.au (B.F.N.); 2 Australian Southern Bluefin Tuna Industry Association, South Quay Blvd, Port Lincoln, SA 5606, Australia; kirstenrough@bigpond.com

**Keywords:** blood fluke, aquaculture, praziquantel, aquatic animal health, molecular diagnostics

## Abstract

Aporocotylid blood flukes *Cardicola forsteri* and *C. orientalis* are an ongoing health concern for the Southern Bluefin Tuna (*Thunnus maccoyii*, SBT) industry, where infections can lead to morbidity and mortality in ranched SBT populations. This study compared blood fluke infection in SBT from two companies during the 2021 ranching season. Both companies administered the same dosage of praziquantel approximately 5 weeks after transfer, feeding with frozen baitfish daily; the only difference in the company’s practices was that the pontoons were located 2.5 km apart. Infection severity was measured as prevalence and intensity by quantifying adult *C. forsteri* in SBT heart and copy numbers of *C. forsteri* and *C. orientalis* ITS-2 DNA in SBT heart and gills. Data from the 2018 and 2019 harvests of SBT were used to make comparisons with 2021 harvest data. *Cardicola orientalis* was detected at transfer and no longer detected after treatment with praziquantel. *Cardicola* spp. were present in 83% of sampled SBT in 2021. Both companies demonstrated similar patterns of infection, and Company A had higher prevalence and intensity of *Cardicola* spp. infection. Based on *C. forsteri* ITS-2 DNA, infection intensity at harvest was significantly greater for both companies in 2021 when compared to 2018 and 2019. Continued monitoring of *Cardicola* spp. in SBT and improvements in diagnostics contribute to our understanding of *Cardicola* spp. epizootiology and the detection of changes in treatment efficacy.

## 1. Introduction

From the genus *Thunnus*, Atlantic Bluefin Tuna (*T. thynnus*, ABT), Pacific Bluefin Tuna (*T. orientalis*, PBT) and Southern Bluefin Tuna (*T. maccoyii*, SBT) are commercially important Bluefin Tuna (BFT) species. *Thunnus* spp. are highly migratory, and the distribution ranges of these three species overlap and span the globe. BFT are valued fish due to their large size and high fat content, making them prized by Japan for sashimi [1]. SBT ranching has been widely used in the industry since 1990 after fishing quotas were introduced [1]. Ranching enabled companies to increase fish weight by 10–20 kg over a 6-month period and maximise their fat content to obtain premium market value on each tuna [1]. Purse seine vessels capture juvenile SBT during their migration through the Great Australian Bight in the Austral summer where they are towed back and then transferred to grow-out pontoons in the Spencer Gulf for approximately 6 months, being fed a daily diet of baitfish before they are harvested [2]. The SBT industry accounted for ~46% of the state’s gross aquaculture production value in 2020/21 [3,4].

The primary health concern of SBT are the aporocotylid blood flukes *Cardicola forsteri* and *C. orientalis* [5,6,7,8,9]. Some members of the genus *Cardicola* are commercially important pathogens, where *C. forsteri* has previously been linked to a mortality event in SBT [10,11] and *C. orientalis* has been identified as a highly pathogenic species in PBT [12,13]. *Cardicola* spp. require both an intermediate and definitive host. Free-living miracidia emerge from the egg and infect the intermediate host, a terebellid polychaete [14,15]. After maturation, free-living cercariae emerge and infect the definitive fish host [15,16]. After infection, adult *C. forsteri* colonise the heart of the host [17], while adult *C. orientalis* are present in the afferent branchial arteries of the gills [12,13,18,19]. Eggs from both species mostly lodge within the lamellae and in the afferent filament arteries of the gills [12]. Gill lesions caused by miracidia hatching from eggs can lead to severe subacute branchitis and mortality in SBT populations [11]. With the potential to cause mass morbidity and mortality, eggs are usually the most destructive stage of the blood fluke life cycle [20,21]. In 2011, the anthelmintic medication, praziquantel (PZQ), was introduced to the SBT industry and is the only known treatment option for the management of blood fluke infections [22]. PZQ is an effective single-dose treatment that is used to break the cycle of parasitic infection by targeting adult *Cardicola* spp. parasitic helminths [23]. The SBT industry administers PZQ treatment by injecting freshly caught baitfish with PZQ, which are then fed to SBT approximately 5 weeks after transfer to ranching pontoons [22,24,25]. Since treating SBT affected by *Cardicola* spp. infection with PZQ, there has been a significant industry-wide reduction of mortalities, from 10–15% to less than 1%. [9,25].

Previous studies have evaluated the infection by *Cardicola* spp. in a single company and comparing two different companies in 2018 and 2019 [9,25]. They have also investigated *Cardicola* spp. infection severity at harvest over a three-year period for seven different companies [26]. Across each of the studies, *C. forsteri* was identified as the dominant species, while *C. orientalis* is rarely detected [9,25,26]. PZQ has maintained high efficacy when treating *Cardicola* spp. 10 years after introduction to the SBT industry when comparing PZQ-treated and -untreated pontoons [9,25,26]. Monitoring investigations provide valuable information on the variability between ranching years, companies and treatment. This study investigated the effect of ranching time on *Cardicola* spp. infection in SBT from Port Lincoln, South Australia. Two commercial companies, not previously reported on during the ranching season, both utilising PZQ treatment since at least 2018, were monitored at four time points over the 2021 ranching season. Historical data from 2018 and 2019 were utilised to investigate the effect of ranching year on *Cardicola* spp. infection severity at harvest [26].

## 2. Materials and Methods

### 2.1. Sample Collection

SBT were sampled from two companies during commercial operations. SBT captured from the Great Australian Bight were first transferred to grow-out sites immediately south of Port Lincoln, South Australia, before being transferred to separate pontoons (33°27′ S, 132°04′ E), where they were fed a daily diet of baitfish (Table 1). Pontoons of Company A and Company B were located within the Spencer Gulf aquaculture zone near Port Lincoln, South Australia. The lease sites were approximately 2.5 km apart, and the approximate depth was 20–25 m [9,27]. Sampling occurred at four time points over the 2021 ranching season during commercial operations: transfer (week 0), pre-treatment (week 4), post-treatment (week 10), and harvest (week 16). Twelve SBT were sampled at transfer (week 0). SBT from Company A were transferred from the tow pontoon on 24 March 2021, and SBT from Company B were transferred on 12 March 2021. SBT in all pontoons were orally administered a PZQ treatment by PZQ-injected baitfish at week 5 at a dose of 30 mg/kg body weight. Table 1 outlines the sample sizes of SBT opportunistically selected for sampling across the remaining three time points observed in the 2021 season. Due to the observational nature of this study, where samples were collected from commercially run pontoons during commercial harvest operations in 100% treated PZQ pontoons, a negative control was not able to be implemented. At times, external factors such as weather conditions or time constraints impacted the attainment of the desired sampling sizes.

Within pontoons, sampled SBT were captured, euthanised and processed using standard industry harvest procedures during commercial harvest operations [9]. Sample collection of SBT hearts and gills followed previously described methods [9,25]. Hearts were placed into a clean plastic tub. A small section (no larger than 0.5 cm^3^) was extracted near the apex of the ventricle from the heart and from the second left gill arch, which were individually fixed in 1.5 mL RNA*later*^®^ (Thermo Fisher Scientific, Scoresby, VIC, Australia). All samples were stored on ice for subsequent DNA extraction. On shore, gilled and gutted weight (kg) and length (cm) of each SBT were recorded. Whole weights were estimated using the following formula: gilled and gutted weight (kg)/0.87. This value as well as SBT length were used to calculate a condition index using the Southern Australia tuna industry formula: whole weight (kg)/length (m^3^). Cumulative mortalities of ranched SBT from transfer to harvest in 2021 were obtained from each company (Table 1).

SBT data for 2018 and 2019 were obtained from [26], with Company A shown as Company G and Company B shown as Company E. SBT samples collected from Company A and Company B at harvest in 2018 and 2019 followed the same collection protocol as samples collected from the 2021 season, and the data were used to compare Companies A and B at harvest over a 3-year period. Table 2 outlines the pontoon characteristics and sample sizes of SBT opportunistically selected for sampling at harvest for the three analysed years. Cumulative mortalities for each harvest year were obtained for the pontoons that the SBT were sampled from (Table 2).

### 2.2. Sample Analysis

SBT hearts and gills were processed using previously described methods [25,28]. Hearts were individually dissected and flushed with water to dislodge adult flukes 2–4 h after sampling. Contents from heart flushes were placed into Petri dishes and observed under a dissecting microscope to count all adult blood flukes.

Genomic DNA was extracted from SBT heart and gill samples using the DNeasy Blood and Tissue Kit (Qiagen, Hilden, Germany). The NanoDrop^TM^ Lite Spectrometer determined that all samples have sufficient concentrations for conducting polymerase chain reactions (PCR), ranging between 27.3 and 1568.4 ng/µL. All samples had acceptable A_260_:A_280_ ratios ranging between 1.86 and 2.15, where ~1.8 is accepted as pure [29]. Extracted DNA was analysed via quantitative PCR (qPCR) to detect and quantify *C. forsteri* and *C. orientalis* [9]. SBT gill samples were also analysed for the presence of *C. opisthorchis* DNA, which has not been reported in SBT to date. Species-specific primers and probes used in this study were designed to target heterogeneous areas of the second internal transcribed spacer (ITS-2) of ribosomal DNA to detect *C. forsteri*, *C. orientalis* and *C. opisthorchis* [5,7].

### 2.3. Statistical Analysis

Effects of time and company on SBT condition index as well as *C. forsteri* and *C. orientalis* infection were analysed using GraphPad Prism 9 (GraphPad software, San Diego, CA, USA). Samples from Company A and Company B were also directly compared at each sampling time point. All statistical analysis assumed significance at *p* ≤ 0.05.

The effect of time in ranching and company on SBT condition index were evaluated using a two-way ANOVA followed by a Tukey’s multiple comparisons test to compare each company and timepoint.

The severity of infection with *Cardicola* spp. in SBT was described by prevalence and mean intensity [30]. Prevalence is determined as the percentage of infected hosts from the sampled population, while mean intensity is defined as the average number of adults or copy number/mg DNA per infected host from the sampled population. Chi-square was used to assess the effect of time on *Cardicola* spp. prevalence during the 2021 ranching season and the effect of year for the data for different harvest years. Fisher’s exact test was used to compare *Cardicola* spp. prevalence between companies at each time point over the 2021 ranching season and between harvest years. Logistic regression was used to determine the relationship between condition index, company, harvest year and weeks in ranching on *Cardicola* spp. infection prevalence. Univariate regression was used to analyse each variable individually, and where *p* < 0.25, variables were compared using multiple logistic regression. To determine the influence of time in ranching and company on *Cardicola* spp. infection intensity in ranched SBT, a two-way ANOVA was used, followed by Tukey’s multiple comparisons test [31]. Spearman’s rank correlation coefficients were used to determine the relationship between *Cardicola* spp. infection intensity and condition index at each time point during the 2021 ranching season. A Brown–Forsythe and Welch one-way ANOVA was performed on pooled company data where time was significant, and company was not a significant factor (*p* > 0.25). A two-tailed McNemar’s x^2^ test was utilised to compare the sensitivity of *C. forsteri* diagnostic methods [32].

## 3. Results

### 3.1. 2021 Season

**Figure 1:**
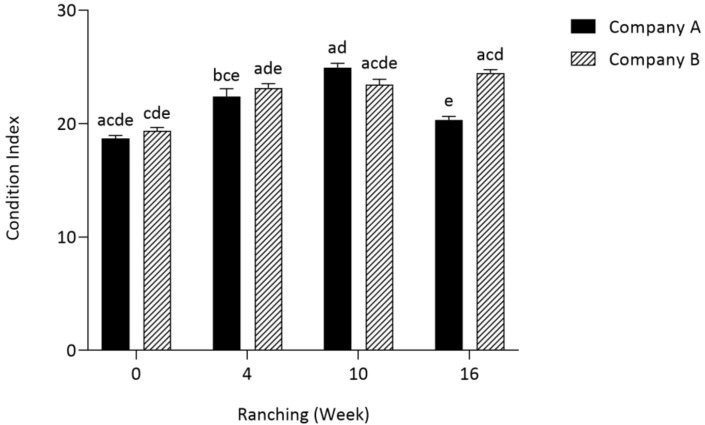
Mean (±SE) condition index of SBT from two commercial companies during 2021 ranching in Port Lincoln, South Australia (*n* = 12 at weeks 0, 4, 10; *n* = 15 at week 16). Companies A and B were PZQ treated at week 5. Different letters denote statistical differences at *p* ≤ 0.05 between companies at each timepoint.

Ranching week (*p* < 0.0001) and company (*p* = 0.0005) both had a significant effect on SBT condition index, and a statistically significant two-way interaction between ranching week and company was present (*p* < 0.0001), showing that the effect of ranching week depended on the company (Figure 1).

Mean condition index of SBT from Company B at week 16 was significantly higher than that from Company A (*p* < 0.0001). No significant differences between companies were seen at any other timepoint.

In Company A, mean condition index was significantly higher at week 4 than week 0 (*p* < 0.0001) and mean condition index was significantly higher at week 10 when compared to week 4 (*p* = 0.0007) and week 16 (*p* < 0.0001). In Company B, mean condition index was significantly higher at week 4 than week 0 (*p* < 0.0001).

The effect of time (weeks in ranching), company (comparison of Companies A and B) and SBT condition index were evaluated for their effect on *Cardicola* spp. prevalence in ranched SBT (Table 3 and Table 4). Time (weeks) significantly increased the likelihood of adult *C. forsteri* in the heart (OR = 1.113, *p* = 0.014). Samples from Company A had a greater likelihood of *C. forsteri* infection in SBT gills (OR = 2.342, *p* = 0.048). SBT condition index had no significant effect on *Cardicola* spp. infection prevalence in SBT.

Company and weeks in ranching were analysed for their effect on *Cardicola* spp. prevalence in SBT (see Supplementary Table S1 for all prevalence results and 95% confidence intervals). In SBT hearts, *C. forsteri* (ITS-2) prevalence was significantly greater in Company A than Company B at week 4 (*p* = 0.0373) and week 10 (*p* = 0.0310); no significant differences were seen at any other time point. There were no significant differences between companies in relation to adult *C. forsteri* in SBT hearts as well as *C. forsteri* (ITS-2) and *C. orientalis* (ITS-2) in SBT gills. Adult *C. forsteri* prevalence in SBT hearts was significantly different between sampling timepoints for Company B (X^2^ = 20.06, d.f. = 3, *p* = 0.0002); however, no significant differences were seen in Company A. Ranching time did not have a significant effect on *C. forsteri* (ITS-2) in SBT hearts and gills.

Ranching time did not have a significant effect on *C. orientalis* (ITS-2) in SBT gills (see Supplementary Table S1 for all prevalence results and 95% confidence intervals). In Company A, *C. orientalis* was detected in one SBT at week 0 (prevalence = 8.33%, CI [0.43–35.39%]) and in three SBT at week 4 (prevalence = 25.00%, CI [8.89–53.23%]) and was not detected in SBT at weeks 10 (prevalence = 0.00%, CI [0.00–24.25%]) and 16 (prevalence = 0.00%, CI [0.00–20.39%]). In Company B, *C. orientalis* was detected in three SBT at week 0 (prevalence = 25.00%, CI [8.89–53.23%]) and in one SBT at week 4 (prevalence = 8.33%, CI [0.43–35.39%]) and was not detected in SBT at weeks 10 (prevalence = 0.00%, CI [0.00–24.25%]) and 16 (prevalence = 0.00, CI [0.00–20.39%]). 

**Figure 2:**
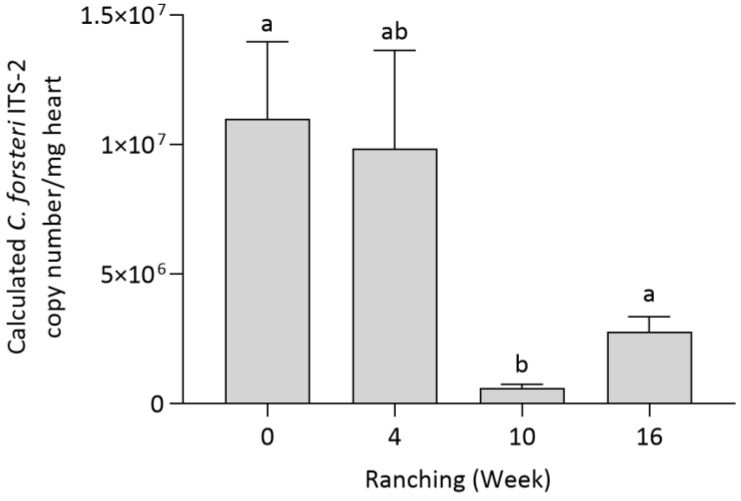
Mean intensity (±SE) of *Cardicola forsteri* in SBT heart from two commercial companies during the 2021 ranching of SBT in Port Lincoln, South Australia (*n* = 14–24 for each timepoint). Different letters denote statistical differences at *p* ≤ 0.05 between companies at each timepoint.

The effect of company and ranching time on *Cardicola* spp. intensity in SBT was analysed (see Supplementary Table S2 for all intensity results and standard error). Ranching week had a significant effect on *C. forsteri* (ITS-2) intensity in SBT hearts (*p* = 0.0127), showing *C. forsteri* (ITS-2) intensity to decline over time. Company did not have a significant effect on *C. forsteri* (ITS-2) intensity in SBT hearts. *Cardicola forsteri* (ITS-2) intensity from Company A and Company B was pooled to compare the effect of each time point. *Cardicola forsteri* (ITS-2) intensity in the heart was significantly higher at week 0 (*p* = 0.0174) and week 16 (*p* = 0.0082) when compared to week 10 (Figure 2). 

Ranching week and company did not have a significant effect on *C. forsteri* (ITS-2) and *C. orientalis* (ITS-2) intensity in SBT gills. The effect of ranching week and company on *C. forsteri* in SBT hearts could not be assessed due to low prevalence. 

A direct comparison of *C. forsteri* detection methods in SBT heart showed that qPCR (ITS-2) was more sensitive than heart flush microscopy (McNemar’s test x^2^, *p* < 0.0001) (Table 5). 

No significant correlations were observed between SBT condition index and *Cardicola* spp. infection intensity at any time point.

### 3.2. Comparison of Harvest Year

The effect of time (weeks in ranching), company (comparison of Companies A and B), SBT condition index, and harvest year were evaluated for their effect on *Cardicola* spp. prevalence in ranched SBT and were found to have no significant effect on *Cardicola* spp. infection prevalence in SBT (see Supplementary Table S3 for all prevalence results and 95% confidence intervals).

**Figure 3:**
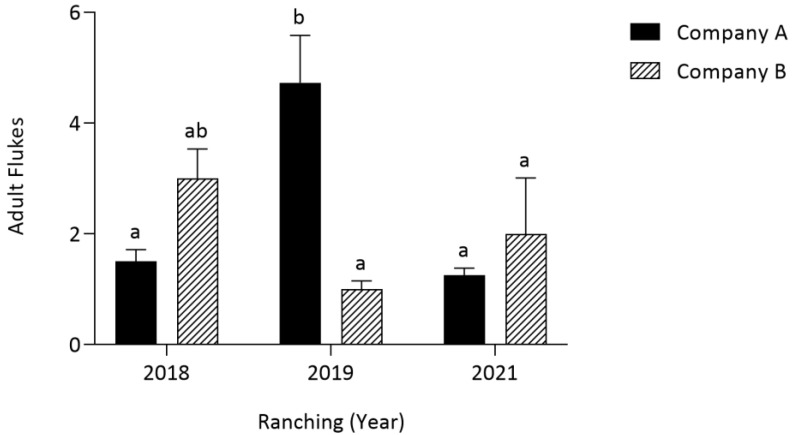
Mean intensity (±SE) of adult *Cardicola forsteri* in SBT heart from two commercial companies at harvest in 2018, 2019 and 2021 in Port Lincoln, South Australia (*n* = 13–15 for each year). Different letters denote statistical differences at *p* ≤ 0.05 between companies at each timepoint.

Company and weeks in ranching were analysed for their effect on *Cardicola* spp. intensity in SBT (see Supplementary Table S4 for all intensity results and standard errors). Ranching year and company did not have a significant effect on adult *C. forsteri* in the heart. However, a statistically significant two-way interaction between ranching year and company was observed (*p* < 0.001). *Cardicola forsteri* number in SBT heart from Company A in 2019 was significantly greater than those in Company A in 2018 (*p* = 0.0015) and 2021 (*p* = 0.0005) and Company B in 2019 (*p* = 0.0002) and 2021 (*p* = 0.0170) (Figure 3).

**Figure 4:**
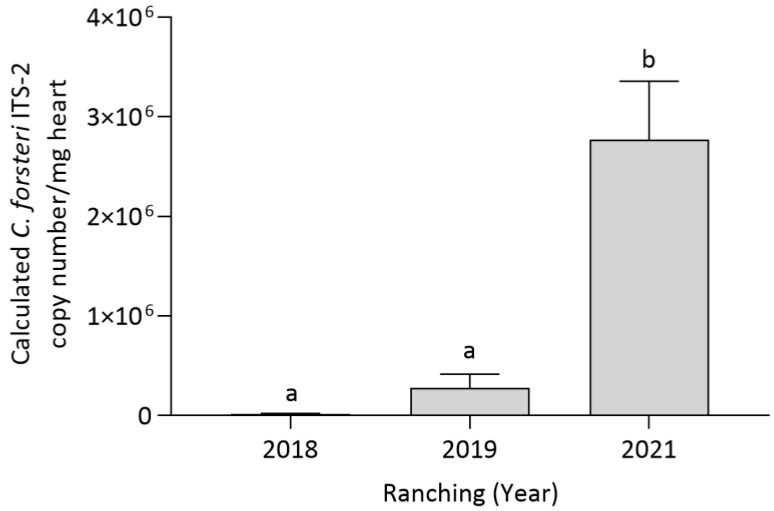
Mean intensity (±SE) of *Cardicola forsteri* (ITS-2) in SBT heart pooled from two commercial companies at harvest in 2018, 2019 and 2021 in Port Lincoln, South Australia (*n* = 15–25 for each year). Different letters denote statistical differences at *p* ≤ 0.05 between companies at each timepoint.

Ranching year had a significant effect on *C. forsteri* (ITS-2) intensity in SBT heart (*p* < 0.0001), showing *C. forsteri* (ITS-2) intensity to increase over time. Company did not have a significant effect on *C. forsteri* (ITS-2) intensity in SBT hearts, and there was no statistically significant two-way interaction between ranching year and company. *Cardicola forsteri* (ITS-2) intensity was pooled from Companies A and B to compare the effect of each time point. *Cardicola forsteri* (ITS-2) intensity in the heart was significantly higher in 2021 when compared to 2018 (*p* = 0.003) and 2019 (*p* = 0.0118) (Figure 4).

**Figure 5:**
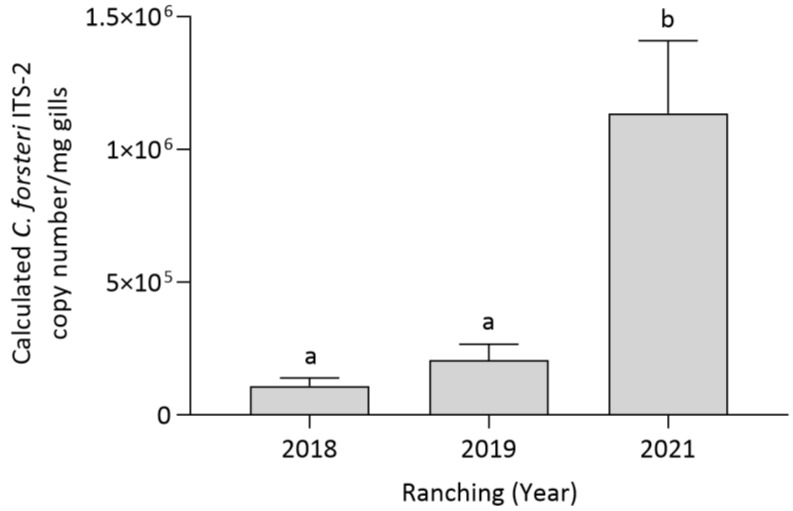
Mean intensity (±SE) of *Cardicola forsteri* (ITS-2) in SBT gills pooled from two commercial companies at harvest in 2018, 2019 and 2021 in Port Lincoln, South Australia (n = 15–25 for each year). Different letters denote statistical differences at *p* ≤ 0.05 between companies at each timepoint.

Ranching year had a significant effect on *C. forsteri* (ITS-2) intensity in SBT gills (*p* < 0.0001), showing *C. forsteri* (ITS-2) intensity to increase over time. Company did not have a significant effect on *C. forsteri* (ITS-2) intensity in SBT gills, and there was no statistically significant two-way interaction between ranching year and company. *Cardicola forsteri* (ITS-2) intensity was pooled from Companies A and B to compare the effect of each time point. *Cardicola forsteri* (ITS-2) intensity in the gills was significantly higher in 2021 when compared to 2018 (*p* = 0.0065) and 2019 (*p* = 0.0137) (Figure 5).

The effect of ranching year and company could not be assessed for *C. orientalis* (ITS-2) in SBT gills as prevalence was low. *Cardicola orientalis* (ITS-2) was only detected in Company A at harvest in 2019, where mean intensity was 1.05 × 10^5^ copy number/mg DNA, and was not detected in harvest gill samples from Company B. 

## 4. Discussion

Time in ranching influenced *Cardicola* spp. infection severity in SBT from both companies, where pontoons were treated with the same single dose of PZQ and had similar patterns of *Cardicola* spp. infection. Based on the count of adult flukes in the heart, two major *C. forsteri* infection peaks were reported prior to the introduction of PZQ, one at 14 and the second at 55 days post transfer [29]. Naturally occurring major infection events were observed early in the ranching season in 2004 [33]. It was proposed that the pattern of infection was caused by successive periods of cercariae shedding from the intermediate hosts occurring approximately 40 to 42 days apart [33]. Although data collected from the 2021 ranching season were from a limited number of time points, a peak infection is proposed between weeks 0 and 4, reflecting the first major infection peak, previously described in ranched SBT. Before PZQ was introduced, a second major infection peak occurred between weeks 4 and 10 [33]. However, our results showed that treatment with PZQ at week 5 resulted in a decline in *C. forsteri* across all assessed organs, preventing the second major infection peak from occurring. The *Cardicola* spp. DNA detected after PZQ treatment can be attributed to not only reinfection but also the presence of eggs. The gills are the sole location of miracidial development, where the eggs remain viable following treatment as they are refractory to PZQ [10,21]. As PZQ targets adult parasitic helminths, *C. forsteri* DNA detected in SBT hearts could be due to the presence of eggs. Miracidia contained within the eggs lodged in heart muscle are not viable and over time are slowly killed by the host immune response [10,20]. It is not currently known how long the eggs remain within SBT hearts; however, *C. chaetodontis* that infect the butterflyfish (Perciformes: Chaetodontidae) in the Great Barrier Reef have been identified as long-lasting indicators of infection, persisting long after declining prevalences of the adult parasite [34]. Both commercial companies in this study have treated all pontoons with PZQ since 2011; hence, the continued management of *Cardicola* spp. in ranched SBT is highly dependent on PZQ maintaining its efficacy. As PZQ is the only known effective treatment for controlling blood fluke infection, a treatment that can also target other life stages of the fluke will greatly benefit the industry [22]. Further understanding on the mechanisms of PZQ efficacy against blood flukes will aid the identification of new drug targets and the development of alternative control measures [35].

Detected in 98.3% of *Cardicola* spp. infections in SBT gills, *C. forsteri* was the most prevalent species in the 2021 ranching season. This species dynamic has been reported since 2013, when ranched SBT gills were first identified to have a greater *C. forsteri* load (100%) in comparison to *C. orientalis* (15.7%) [7]. Prior to 2011 and the introduction of PZQ, *C. orientalis* was retrospectively identified as the dominant species infecting 86% of samples from ranched SBT between 2008 and 2012; however, it is now rarely detected [5,7,9,25]. *Cardicola orientalis* was not detected after PZQ treatment at week 5 and has been rarely detected in SBT at harvest since 2013 [7,9]. As the hosts of *Cardicola* spp. are both cosmopolitan species, the prevalence of *Cardicola* spp. is linked to the distribution of their hosts, either related to the migration of Bluefin Tuna or polychaete abundance [36,37]. The reduction in *C. orientalis* infection severity coincided with the introduction of PZQ, and it is unknown whether the introduction of PZQ caused the continued low infection severity of *C. orientalis* in SBT as the pharmacokinetics of PZQ against *C. orientalis* is not fully understood [21,22]. The low prevalence and intensity of *C. orientalis* in the present study was consistent with a previous survey undertaken shortly after PZQ was introduced, when low prevalence of *C. orientalis* was seen after treatment at harvest in 2013 and it was not detected after treatment in 2014 or 2015 [7]. However, the *C. forsteri* (ITS-2) intensity was greater in 2021 when compared to historical harvest data from 2018 and 2019 for both Company A and Company B. In both heart and gill samples, intensity increased over the three harvest years. Unfortunately, data from the 2020 SBT harvest were not obtained due to COVID-19 restrictions. This means that we cannot confirm whether there is an increasing trend of *C. forsteri* at harvest over the past 4 years or if the results were due to annual variability. However, the intensities observed in recent years are greater than when PZQ was first introduced [7]. Widespread resistance to PZQ has not currently been seen in fish blood flukes, nor have schistosomes and other flatworm infections in humans and other mammals [23]. Induced reductions of PZQ sensitivity have been observed in laboratory experiments with *Schistosoma* and have also been reported from the field and other species unrelated to schistosomes (reviewed in [23]). However, in aquaculture, there has only been one recent report of reduced PZQ efficacy when treating salmon for *Eubothrium* infections in Norway, which caused major losses [38,39,40]. Currently, there is an over-reliance on PZQ as it is the only available treatment option for *Cardicola* spp. in SBT, meaning there is no alternative if efficacy declines or resistance develops. Continued monitoring of *Cardicola* spp. in SBT over successive years provides important epizootiological data to observe and determine changes in species dynamics, especially regarding the continued efficacy of PZQ.

This study was the first to detect *C. orientalis* (ITS-2) DNA in SBT gills at transfer (week 0). However, as qPCR detection cannot distinguish differences in *Cardicola* spp. life stages, it is unknown which *C. orientalis* life stage was present when detected, meaning that SBT may have been infected prior to arrival or newly infected near the site of transfer. As the assay is very sensitive [5], even a low concentration of *Cardicola* spp. DNA will result in positive detection of the parasite. When compared to wild SBT, ranched SBT have a higher *Cardicola* spp. Load, suggesting that the intermediate hosts required to complete the blood fluke life cycle are in proximity to aquaculture sites [16,18]. *Cardicola forsteri* utilises two different terebellid species that are taxonomically close to one another, *Longicarpus modestus* in Australia and *Neomphitrite vigintipes* in Japan [16,41,42]. For *C. orientalis*, the intermediate host was identified in Japan as *Nicolea gracilibranchis*; however, the intermediate host for this species is yet to be identified in Australia [41]. A third species known to infect BFT is *C. opisthorchis*. *Cardicola opisthorchis* infects PBT and ABT alongside *C. forsteri* and *C. orientalis*; however, it has not been reported in SBT to date. All gill samples were screened via qPCR for *C. opisthorchis*, but it was not detected in this study and is consistent with previous observations [7,25].

Both time and company are significant factors when observing SBT condition in 2021. In both companies, SBT condition increased over time; however, at harvest during week 16, the condition index of SBT in Company B was significantly greater than that of Company A. It is unclear why this was the case. It may be due to variabilities in SBT sizes or sample sizes when sampling. Although this study did not find *Cardicola* spp. prevalence and intensity to affect SBT condition, the intensity of *Cardicola* spp. DNA in SBT gills has recently been found to have a significant negative relationship with the condition index [26].

It is difficult to explain why cumulative mortality was greater in Company B when compared to Company A as the cause of SBT death was unknown. However, prior to the introduction of PZQ in 2011, high mortalities of ranched SBT coincided with high *C. orientalis* infection prevalence in SBT samples [5,11,43]. In a study of PBT, *C. orientalis* was identified as the more pathogenic species when compared to *C. forsteri* and *C. opisthorchis*, as they produce a significantly greater number of eggs [6,21]. The accumulation of eggs in the gills or a high *C. orientalis* infection intensity has since been identified to be associated with increased mortalities [13]. In this study, *C. orientalis* infection severity did not appear to be the cause of the difference in cumulative mortalities seen between companies.

Both molecular and microscopic diagnostic methods were used to assess *Cardicola* spp. infection in SBT. Molecular diagnostic methods (qPCR) were more sensitive compared to adult fluke counts for the detection of *C. forsteri* in SBT heart. This information further confirms qPCR as the gold standard for *Cardicola* spp. Detection, where the increased sensitivity provides a greater understanding of *Cardicola* spp. infection severity across all life cycle stages, detecting subtle changes in infections [9,25]. However, qPCR requires specialised equipment, training and laboratory access to be conducted and is therefore not readily accessible to the SBT industry. In comparison, the adult *C. forsteri* count in a heart flush is inexpensive and simple to perform. However, microscopy requires training to identify blood flukes and is a time-consuming process requiring fresh samples, making it logistically difficult to perform for a high number of fish [25,28]. There is a need for an accessible field-based diagnostic method that can assess *Cardicola* spp. infection at the point of care. Recently, a recombinase polymerase amplification lateral flow (RPA-LF) assay was developed for the detection of *Cardicola* spp. in SBT DNA [44]. RPA-LF is a cost-effective and accessible diagnostic method that will be capable of processing large numbers of samples onsite and thus inform management strategies faster than current diagnostic methods.

## Figures and Tables

**Table 1:** Sampling information including pontoon characteristics and SBT sampled at each time point from transfer to harvest in 2021. Both companies treated with PZQ at week 5 of ranching.

	Pre-Treatment (Week 4)	Post-Treatment (Week 10)	Harvest (Week 16)	Cumulative Mortality (%)
**Company A**				
Pontoon 1	6	0	15	0.54
Pontoon 2	0	12	0	0.35
Pontoon 3	6	0	0	0.60
**Company B**				
Pontoon 1	4	11	0	3.00
Pontoon 2	4	1	15	3.00
Pontoon 3	4	0	0	2.00

**Table 2:** Sampling information including pontoon characteristics and ranched SBT sampled at harvest in 2018, 2019 and 2021. Companies treated with PZQ at week 5 of ranching (*n* = 15 in each company at each year).

	Transfer Date	Praziquantel Treatment Dose (mg/kg)	Weeks in Ranching	Cumulative Mortality (%)
**Company A**				
2021	24 March	30	16	0.54
2019	1 March	30	21	0.52
2018	27 February	30	20	0.08
**Company B**				
2021	12 March	30	17	3.00
2019	23 March	18	17	0.27
2018	8 March	42	18	2.33

**Table 3:** Variables assessed for their effect on *Cardicola* spp. prevalence in ranched SBT using simple logistic regression. Bold numbers denote statistical differences at *p* ≤ 0.05 (OR = odds ratio; Z = regression coefficient).

	Adult *C. forsteri* Heart			*C. forsteri* (ITS-2) Heart			*C. forsteri* (ITS-2) Gills			*C. orientalis* (ITS-2) Gills		
	OR	Z	*p*	OR	Z	*p*	OR	Z	*p*	OR	Z	*p*
SBT Condition Index	1.129	1.265	0.206	0.970	0.327	0.744	0.886	1.519	0.129	0.834	1.218	0.223
Company	1.330	0.588	0.556	0.196	2.927	**0.003**	0.406	2.186	**0.029**	1.000	<0.001	>0.999
Time (weeks)	1.118	2.665	**0.008**	1.043	1.082	0.279	0.940	1.907	0.057	0.779	2.347	**0.019**

**Table 4:** Variables assessed for their effect on *Cardicola* spp. prevalence in ranched SBT using multiple logistic regression. Bold numbers denote statistical differences at *p* ≤ 0.05 (OR = odds ratio; Z = regression coefficient). n.a.–not applicable.

	Adult *C. forsteri* Heart			*C. forsteri* (ITS-2) Gills			*C. orientalis* (ITS-2) Gills		
	OR	Z	*p*	OR	Z	*p*	OR	Z	*p*
SBT Condition Index	1.042	0.390	0.696	1.032	0.337	0.736	1.325	1.089	0.276
Company	n.a.	n.a.	n.a.	0.427	1.979	**0.048**	n.a.	n.a.	n.a.
Time (weeks)	1.113	2.465	**0.014**	1.057	1.528	0.126	0.659	1.890	0.059

**Table 5:** Comparison of diagnostic methods for detecting *C. forsteri* within the heart of the same individual SBT (McNemar’s test x^2^, *p* < 0.0001).

	qPCR: *Cardicola forsteri* (ITS-2)			
		+	−	Total
Heart Flush: Adult *Cardicola forsteri*	+	19	3	22
	−	56	20	76
	Total	75	23	98

## Data Availability

The data presented in this study are available in Supplementary Materials.

## Supplementary Materials

The following supporting information can be downloaded at https://www.mdpi.com/article/10.3390/pathogens12121443/s1, Table S1: Prevalence (P) (95% confidence interval) of *Cardicola* spp. in ranched SBT from Port Lincoln, South Australia in 2021; Table S2: Mean intensity (I) (±SE) of *Cardicola* spp. in ranched SBT from Port Lincoln, South Australia in 2021; Table S3: Prevalence (P) (95% confidence interval) of *Cardicola* spp. in ranched SBT from Port Lincoln, South Australia at harvest in 2018, 2019 and 2021; Table S4: Mean Intensity (I) (±SE) of *Cardicola* spp. in ranched SBT from Port Lincoln, South Australia at harvest in 2018, 2019 and 2021.
