# Supplementary material for: Dynamics of Cardicola spp. Infection in Ranched Southern Bluefin Tuna: First Observation of C. orientalis at Transfer
Source: Pathogens. 2023 Dec 13;12(12):1443. doi: 10.3390/pathogens12121443 (PMC10747332; doi:10.3390/pathogens12121443)
Supplement: Supplementary file 1 [file pathogens-12-01443-s001.zip › Supplementary Table S1.pdf]

Supplementary Table S1. Prevalence (P) (95% confidence interval) of *Cardicola* spp. in ranched SBT from Port Lincoln, South Australia in 2021.

	**Adult *C. forsteri* heart**		***C. forsteri* (ITS-2) heart**		***C. forsteri* (ITS-2) gills**		***C. orientalis* (ITS-2) gills**	
	n	P (%)	n	P (%)	n	P (%)	n	P (%)
**COMPANY A**								
Week 0	12	8.33 (0.43-35.59)	12	75.00 (46.77-91.11)	12	83.33 (55.20-97.04)	12	8.33 (0.43-35.39)
Week 4	12	41.67 (19.33-68.05)	12	100.00 (75.75-100.00)	12	83.33 (55.20-97.04)	12	25.00 (8.89-53.23)
Week 10	12	0.00 (0.00-24.25)	10	90.00 (59.59-99.49)	12	58.33 (31.95-80.67)	12	0.00 (0.00-24.25)
Week 16	15	26.67 (10.90-51.95)	15	93.33 (70.18-99.66)	15	53.33 (30.12-75.19)	15	0.00 (0.00-20.39)
**COMPANY B**								
Week 0	12	8.33 (0.43-35.39)	12	66.67 (39.06-86.19)	12	58.33 (31.95-80.67)	12	25.00 (8.89-53.23)
Week 4	12	16.67 (2.96-44.80)	12	58.33 (31.95-80.67)	12	50.00 (25.39-74.62)	12	8.33 (0.43-35.39)
Week 10	12	0.00 (0.00-24.25)	12	41.67 (19.33-68.05)	12	33.33 (13.81-60.94)	12	0.00 (0.00-24.25)
Week 16	13	69.23 (42.37-87.32)	13	84.62 (57.77-97.27)	15	46.67 (24.81-69.88)	15	0.00 (0.00-20.39)
