# Supplementary material for: Dynamics of Cardicola spp. Infection in Ranched Southern Bluefin Tuna: First Observation of C. orientalis at Transfer
Source: Pathogens. 2023 Dec 13;12(12):1443. doi: 10.3390/pathogens12121443 (PMC10747332; doi:10.3390/pathogens12121443)
Supplement: Supplementary file 1 [file pathogens-12-01443-s001.zip › Supplementary Table S2.pdf]

Supplementary Table S2. Mean intensity (I) (±SE) of *Cardicola* spp. in ranched SBT from Port Lincoln, South Australia in 2021.

	Adult *C. forsteri* heart		*C. forsteri* (ITS-2) heart		*C. forsteri* (ITS-2) gills		*C. orientalis* (ITS-2) gills	
	n	I	n	I	n	I	n	I
**COMPANY A**								
Week 0	12	2.00 (±0.00)	12	9.73×10^6 ($\pm 3.01 \times 10^6$)	12	1.89×10^6 ($\pm 6.65 \times 10^5$)	12	1.65×10^5 (±0.00)
Week 4	12	2.80 (±0.92)	12	1.04×10^7 ($\pm 5.65 \times 10^6$)	12	9.20×10^6 ($\pm 6.06 \times 10^6$)	12	1.69×10^4 ($\pm 1.23 \times 10^4$)
Week 10	12	0	10	6.11×10^5 ($\pm 1.67 \times 10^5$)	12	8.23×10^5 ($\pm 2.65 \times 10^5$)	12	0
Week 16	15	1.25 (±0.25)	15	2.69×10^6 ($\pm 9.13 \times 10^5$)	15	1.22×10^6 ($\pm 4.78 \times 10^5$)	5	0
**COMPANY B**								
Week 0	12	1.00 (±0.00)	12	1.24×10^7 ($\pm 5.56 \times 10^6$)	12	9.82×10^5 ($\pm 6.66 \times 10^5$)	12	1.87×10^6 ($\pm 1.66 \times 10^6$)
Week 4	12	1.00 (±0.00)	12	8.98×10^6 ($\pm 4.03 \times 10^6$)	12	2.87×10^6 ($\pm 1.01 \times 10^6$)	12	3.83×10^5 (±0.00)
Week 10	12	0	12	6.05×10^5 ($\pm 2.56 \times 10^5$)	12	1.36×10^6 ($\pm 5.09 \times 10^5$)	12	0
Week 16	13	2.66 (±1.21)	13	2.87×10^6 ($\pm 7.15 \times 10^5$)	15	1.04×10^6 ($\pm 2.57 \times 10^5$)	15	0
