# Supplementary material for: Dynamics of Cardicola spp. Infection in Ranched Southern Bluefin Tuna: First Observation of C. orientalis at Transfer
Source: Pathogens. 2023 Dec 13;12(12):1443. doi: 10.3390/pathogens12121443 (PMC10747332; doi:10.3390/pathogens12121443)
Supplement: Supplementary file 1 [file pathogens-12-01443-s001.zip › Supplementary Table S3.pdf]

Supplementary Table S3. Prevalence (P) (95% confidence interval) of *Cardicola* spp. in ranched SBT from Port Lincoln, South Australia at harvest in 2018, 2019 and 2021.

	**Adult *C. forsteri* heart**		***C. forsteri* (ITS-2) heart**		***C. forsteri* (ITS-2) gills**		***C. orientalis* (ITS-2) gills**	
	n	P (%)	n	P (%)	n	P (%)	n	P (%)
**COMPANY A**								
2021	15	26.67 (10.90-51.95)	15	93.33 (70.18-99.66)	15	53.33 (30.12-75.19)	15	0.00 (0.00-20.39)
2019	15	73.33 (48.05-89.10)	15	86.67 (62.12-97.63)	15	86.67 (62.12-97.63)	15	13.33 (2.37-37.88)
2018	15	40.00 (19.82-64.25)	15	66.67 (41.71-84.82)	13	61.54 (35.52-82.29)	13	0.00 (0.00-20.39)
**COMPANY B**								
2021	13	69.23 (42.37-87.32)	13	84.62 (57.77-97.27)	15	46.67 (24.81-69.88)	15	0.00 (0.00-20.39)
2019	15	20.00 (7.05-45.19)	11	18.18 (3.23-47.70)	15	80.00 (54.82-92.95)	15	0.00 (0.00-20.39)
2018	14	50.00 (26.80-73.20)	14	85.71 (60.06-97.46)	15	80.00 (54.82-92.95)	15	0.00 (0.00-20.39)
