# Supplementary material for: Dynamics of Cardicola spp. Infection in Ranched Southern Bluefin Tuna: First Observation of C. orientalis at Transfer
Source: Pathogens. 2023 Dec 13;12(12):1443. doi: 10.3390/pathogens12121443 (PMC10747332; doi:10.3390/pathogens12121443)
Supplement: Supplementary file 1 [file pathogens-12-01443-s001.zip › Supplementary Table S4.pdf]

Supplementary Table S4. Mean Intensity (I) (±SE) of *Cardicola* spp. in ranched SBT from Port Lincoln, South Australia at harvest in 2018, 2019 and 2021.

	**Adult *C. forsteri* heart**		***C. forsteri* (ITS-2) heart**		***C. forsteri* (ITS-2) gills**		***C. orientalis* (ITS-2) gills**	
	n	I	n	I	n	I	n	I
**COMPANY A**								
2021	15	1.25 (±0.25)	15	2.69×10^6 ($\pm 9.13 \times 10^5$)	15	1.22×10^6 ($\pm 4.78 \times 10^5$)	15	0
2019	15	4.73 (±1.00)	15	3.19×10^5 ($\pm 1.56 \times 10^5$)	15	3.86×10^5 ($\pm 9.29 \times 10^4$)	15	1.05×10^4 ($\pm 1.50 \times 10^3$)
2018	15	1.50 (±0.34)	15	2.59×10^4 ($\pm 6.62 \times 10^3$)	13	6.36×10^4 ($\pm 2.36 \times 10^4$)	13	0
**COMPANY B**								
2021	13	2.67 (±1.21)	13	2.87×10^6 ($\pm 7.14 \times 10^5$)	15	1.04×10^6 ($\pm 2.57 \times 10^5$)	15	0
2019	15	1.67 (±0.58)	11	1.12×10^4 ($\pm 6.39 \times 10^2$)	15	1.22×10^4 ($\pm 2.66 \times 10^3$)	15	0
2018	14	3.00 (±0.76)	14	9.58×10^3 ($\pm 6.87 \times 10^3$)	15	1.39×10^5 ($\pm 4.72 \times 10^4$)	15	0
